# Morin Inhibits Proliferation of SW480 Colorectal Cancer Cells by Inducing Apoptosis Mediated by Reactive Oxygen Species Formation and Uncoupling of Warburg Effect

**DOI:** 10.3389/fphar.2017.00640

**Published:** 2017-09-12

**Authors:** Thomas Sithara, K. B. Arun, H. P. Syama, T. R. Reshmitha, P. Nisha

**Affiliations:** ^1^Agro Processing and Technology Division, National Institute for Interdisciplinary Science and Technology (CSIR) Thiruvananthapuram, India; ^2^Academy of Scientific and Innovative Research New Delhi, India

**Keywords:** colorectal cancer, reactive oxygen species, apoptosis, Warburg effect, energetic stress

## Abstract

The study under investigation focuses on *in vitro* antiproliferative efficacy of the flavonoid morin and the mechanisms by which it inhibits the growth of colon cancer using SW480 colon cancer cells with emphasis on Warburg effect. It was found that the cell proliferation was significantly inhibited by morin in a dose and time dependent manner. Morin induced apoptosis that was correlated with increased levels of reactive oxygen species formation and loss of mitochondrial membrane potential of the cells. In addition, an increase in cleaved PARP, cleaved caspase 3, cleaved caspase 8, cleaved caspase 9 and Bax as well as a decrease in Bcl 2 was observed, indicating morin is inducing both intrinsic as well as extrinsic pathway of apoptosis. This was further confirmed by using downstream caspase 3 inhibitor which indicated that caspase 3 inhibition reduces morin induced cell death. Moreover, the impact of morin on over all energy status when determined in terms of total cellular ATP level showed a decline with low level of glucose uptake and Glut1 expression. The results indicate that morin exerts antiproliferative activity by inducing apoptosis and by reducing Warburg effect in the evaluated cell lines and provide preliminary evidence for its anticancer activity.

## Introduction

Cancer is one of the major diseases with high mortality rate and has become the major cause of death among the population in both developing and developed countries (Jemal et al., 2011). Among various types of cancers, colorectal cancer is the third most commonly diagnosed cancer after lung and breast cancers and the fourth most common cause of death (Parkin et al., 2002; World Cancer Research Fund and American Institute for Cancer Research, 2007; Spanos et al., 2008) accounting for over 10% of all cancer incidence and almost 8% of total cancer deaths (Center et al., 2011). The incidence of CRC has increased steadily in recent years and it is predicted that by the year of 2035 worldwide the number of CRC cases will rise to 1.36 million for men and 1.08 million for women (Stewart and Wild, 2014).

Epidemiological data suggests that diet plays a major role in the prevention and management of CRC (Bishehsari et al., 2014). It is reported that 90% of the CRC mortality is attributed to the dietary factors (Doll and Peto, 1981) and daily consumption of >400 g/day of vegetables and fruits is reported to reduce the risk of CRC by 40% (Willett, 1995). Fruits and vegetables are the rich source of dietary fiber and bioactive compounds and most of the plant bioactives are reported to bind to the dietary fiber. Due to colonic fermentation of the dietary fiber by the probiotics in the region, these bioactives are getting released in the colon. These plant-derived dietary substances can play a vital role in cancer prevention by blocking the action of carcinogens on target tissue thereby suppressing the cancer development. These phytochemicals are also reported to play a significant role in the secondary prevention, by reduction of cell growth or enhancement of differentiation and apoptosis in tumor initiated cells (Surh, 2003).

As the apoptotic pathways are altered significantly in cancer cells compared to normal cells, it has been used as a therapeutic window for the development of useful anticancer drugs. The malignant transformation of a normal cell into a cancer cell is also accompanied by many metabolic alterations like high glucose uptake to meet the energy requirement of the tumor via glycolytic pathway (Warburg, 1956). The high glucose requirement in cancer cells is fulfilled by upregulating transmembrane glucose transporters; catalyze facilitative diffusion of glucose into the cells (Hauptmann et al., 2005). Out of this, Glucose transporter 1 (Glut 1) is regarded as a master regulator (Zhang et al., 2013). Activation of many signaling pathways (Ras, PI3K/Akt, and c-Myc) will further increase gene expression of Glut 1 and which intern facilitates glucose importation (Fritz and Fajas, 2010). Therefore, glucose transporters are targeted to block glucose regulated metabolism as a therapeutic approach for oncogenic progression.

Studies have explored the anticancer properties of extracts and phytochemicals from a large number of fruits and vegetables (Karikas, 2010; Saunders and Wallace, 2010). Protective elements in them include selenium, vitamins, food polyphenols, such as flavonoids, phytoalexins, phenolic acids, indoles, carotenoids, etc. (Surh, 2003; Russo, 2007). Curcumin, epigallocatechin gallate, quercetin, kaempferol, catechin, lycopene, resveratrol and naringin are few compounds coming under this group and studied widely (Mukhopadhyay et al., 2001; Aggarwal et al., 2004; Ackland et al., 2005; Hwang and Lee, 2006; Bishayee, 2009; Wong and Fiscus, 2015). In-depth research on these bioactive compounds revealed their ability to exert their antineoplastic activities (Tarapore et al., 2012) and led to the emergence of alternate forms of cancer treatment approach called nutrition therapy, to fight against cancer through a healthy diet while presenting none of the side-effects which are often encountered by patients undergoing treatment. Thus, identifying these bioactive molecules, evaluating their broad range pharmaceutical activity, evaluating their precise mechanism of action could abet in the treatment of cancer.

Various parts of Moraceae family of plants like mulberry, figs and Chinese herbs are being used against different types of diseases including inflammation, cardiovascular diseases and cancers like breast cancer, colon cancer and cervical cancer (Patel and Patel, 2011; Fathy et al., 2013; Qadir et al., 2014) and epidemiological studies report that adequate intakes of flavonoid-rich foods can reduce the risk of coronary heart disease and certain types of cancer (Yang et al., 2001). The present study focused on morin, (3,5,7,20,40-pentahydroxyflavone) a flavonoid mainly found in members of the Moraceae family and also in leaves of common guava, onion, almond, etc (Kawabata et al., 1999; Brown et al., 2003). Park et al. (2014) showed that morin can induce apoptosis in human leukemic cells. Kawabata et al. (1999) showed that morin exerted protective effect against chemically induced rat tongue carcinogenesis and Hsiang et al. (2005) has reported that morin suppressed phorbol ester-induced transformation of hepatocytes. Another study reported the inhibition of azoxymethane-induced aberrant crypt foci in rats by morin (Tanaka et al., 1999). However, very little is known about the mechanism of anticancer property of morin against CRC. Study by Hyun et al. (2015) reported the anticancer activity of morin against CRC in HCT 116 cells and found that morin could induce apoptosis mediated by ROS formation.

Alterations in glucose uptake and glycolytic flux (Warburg effect) are observed in colon and pancreatic tumors and the malignant cells use this metabolic pathway for generating ATP, the main source of their energy supply which make these tumors highly resilience to chemotherapy (Bryant et al., 2014). Recent studies emphasize the importance of modulation of Warburg effect that may help to overcome resistance to treatment strategies in such cancers (Aguilera et al., 2016). The available reports suggest the anticancer potential of morin, however, its mechanism is not fully established. As increased aerobic glycolysis is seen in most of the human cancers and if morin can modulate the Warburg effect that can have broad therapeutic applications which can further substantiate its anticancer efficacy. Therefore, the present study is designed to validate the potential of morin for colon cancer therapies based on apoptosis and energetic stress using SW480 colon cancer cells.

## Materials and Methods

### Materials

Dulbecco’s modified eagle’s media (DMEM), antibiotic-antimycotic mix, MTT (3-(4, 5-dimethylthiazol-2-yl)-2, 5-diphenyl tetrazolium bromide), 2′,7′-dichlorofluorescin-diacetate (DCFH-DA), Rhodamine 123 (Rh123), Hoechst 33342, morin and glutaraldehyde were purchased from Sigma–Aldrich Chemicals (St Louis, MO, United States). Fetal bovine serum (FBS) was purchased from Gibco-BRL (Auckland, New Zealand). Annexin V – FITC assay kit (600300) was purchased from Cayman chemicals. Glutathione colorimetric assay kit (K261-100) and catalase activity assay kit (K773-100) were purchased from Biovision. BCA protein assay kit was procured from Pierce Biotechnology, Rockford, IL, United States. Caspase 3 specific inhibitor, z-DEVAD-fmk was purchased from R&D Systems (Minneapolis, MN, United States). Primary antibodies (β actin, cleaved PARP, cleaved caspase 3, cleaved caspase 8, cleaved caspase 9, Bcl 2, Bax and Glut 1) and corresponding secondary antibodies for western blot analysis were purchased from Santa Cruz Biotechnology, United States. Clarity Western ECL substrate was purchased from Bio-rad, United States. 2-(7-Nitrobenz-2-oxa-1,3-diazol-4-yl) amino-2-deoxy-D-glucose (2-NBDG) was obtained from Molecular Probe (Invitrogen Life Technologies, Carlsbad, CA, United States). All other chemicals used were of the standard analytical grade.

### Cell Culture and Treatment

The human colon cancer cells (SW480) were obtained from ATCC (American Type Culture Collection, Manassas, United States) were maintained in DMEM supplemented with 10% FBS, 1% antibiotic–antimycotic mix at 37°C under a humidified 5% CO_2_ and 95% air atmosphere. Cells were exposed to 0.25% trypsin-EDTA and harvested cells were seeded at a density of 1 × 10^4^ cells/well on 24 well-plates, 6-well plates (Costar, United States) and 96-well black plates (BD Biosciences, Franklin Lakes, NJ, United States) for different assays.

### Cell Viability by MTT Assay

The viability of SW480 cells was assessed by 3-(4,5-dimethylthiazol-2-yl)-2,5-diphenyltetrazolium bromide (MTT) reduction assay as described previously (Mosmann, 1983). Briefly, after treating the cells with different concentrations of morin, (ranging from 50 to 500 μM) for 24 and 48 h, washed and MTT (0.5 g/L) was added to each well, incubated at 37°C in a CO_2_ incubator. After 4 h incubation, 10% SDS in DMSO was added to each well, and the absorbance of solubilized MTT formazan products was measured at 570 nm after 45 min using a microplate reader (BIOTEK-United States). Percentage of cytotoxicity was calculated using the following equation. Results were expressed as % cell viability.


$$Percentage\,\;of\,\;Toxicity=\frac{Absorbance\,\;of\,\;Control-Absorbance\,\;of\,\;Sample}{Absorbance\,\;of\,\;Control}\times 100$$

Cell viability (%) = 100 – percentage toxicity.

### Scanning Electron Microscope (SEM) Observation

The morphological changes on treatment with morin were observed using SEM. SW480 cells were treated with morin (150, 200, and 250 μM) and camptothecin (50 μM) for 48 h and cells in each group were fixed in phosphate-buffered 2.5% glutaraldehyde solution. After that cells were rinsed in PBS three times and dehydrated by ascending ethanol series (50, 70, 80, 90, 95, and 100%) and samples were stuck on to Aluminum stub with the help of a carbon tape, sputter coated with gold–palladium to render them electrically conductive by using HUMMLE VII Sputter Coating Device (Anatech Electronics, Garfield, NJ, United States). The micrographs were taken using SEM, JEOL JSM-5600LV (Italy) at magnification of 1500× and 5000×.

### Nuclear Staining Using Hoechst 33342 Stain

The changes in nuclear morphology of the cells on treatment with morin (150, 200, and 250 μM) and camptothecin (50 μM) for 48 h were examined using the cell-permeable DNA dye, Hoechst 33342 (Hickman, 1992). After incubation with morin, cells were stained with Hoechst 33342 (10 μg/mL) for 20 min at 37°C followed by washing with PBS for 3 times and the nuclei were observed under fluorescent microscope (Pathway 855, BD Bioscience, United States) equipped with filters in the excitation of 350 nm and emission of 460 nm.

### Cell Apoptotic Analysis by Flow Cytometry

Cell apoptosis was measured using Annexin V-fluorescein isothiocyanate (FITC)/propidium iodide staining. Briefly, cells were incubated with varying concentrations of morin (150, 200, and 250 μM) and camptothecin (50 μM) for 48 h. Treated cells were collected by trypsinization and centrifuged at 2300 rpm for 5 min, washed with cold PBS and resuspended in diluted binding buffer. It was mixed well, centrifuged at 400 × *g* for 5 min. The supernatant was discarded and cells were stained with a mixture of FITC-Annexin-V (10 μl) and propidium iodide solution (10 μl) in binding buffer (5 ml), from the Annexin-V apoptosis detection kit (Cayman Chemical Company, United States) and incubated for 10 min at room temperature in the dark condition, centrifuged at 400 × *g* for 5 min and then re-suspended in 1 ml assay binding buffer and analyzed by Fluorescence Activated Cell Sorting (BD FACS Aria II, BD Biosciences, United States) within 1 h following the staining. The data acquisition and analysis were performed using BD FACSDiva^TM^ Software v6.1.2 and a minimum of 10000 cells was analyzed in each group. The ratio of apoptotic cells was measured by flow cytometry as described by manufacturer’s instructions.

### Measurement of Mitochondrial Membrane Potential (ΔΨm)

The effect of morin on the mitochondrial membrane potential was identified by staining with Rhodamine 123, a cationic fluorescent indicator which selectively accumulates within the mitochondria in a membrane potential dependent way (Zhang et al., 2008). Cells grown in 6 well plates were treated with indicated concentration of morin (150, 200, and 250 μM) for 24 and 48 h and positive control, H_2_O_2_ (200 μM) for 2 h. The harvested cells were rinsed twice with PBS, resuspended in Rh123 (0.625 mg/ml) and incubated at 37°C for 25 min in the dark followed by rinsing with several changes of PBS. The fluorescence was detected by Fluorescence Activated Cell Sorting (BD FACS Aria II, BD Biosciences, United States). A reduction in green rhodamine 123 fluorescence indicates reduced ΔΨm. The data acquisition and analysis were performed using BD FACSDiva^TM^ Software v6.1.2, and a minimum of 10000 cells was analyzed from each group.

### Immunoblot Analysis

Following incubation of cells with morin (150, 200, and 250 μM) and camptothecin (50 μM) for 48 h, cells were washed twice with ice cold PBS, lysed in ice-cold lysis buffer (50 mM Tris–HCl, 150 mM sodium chloride, 0.5 mM EDTA, 0.1% sodium dodecyl sulfate, 1% Triton X-100 and protease inhibitor cocktail, pH 8.0) for 30 min on ice and were centrifuged at 12000 × *g* for 10 min. The protein content of the lysate was measured using BCA protein assay kit. Lysates were diluted to an equal concentration of total protein and supernatants were then stored at -80°C until analysis. These samples were boiled for 10 min at 75°C in reducing sample buffer (62.5 mM Tris–HCl pH6.8, 2% SDS, 10% glycerol, 5% β-mercaptoethanol and 0.01% bromophenol blue). The lysate containing 50 μg of protein was subjected to SDS–PAGE on 12% gel and transferred onto a polyvinylidene difluoride membrane (Immobilon P^TM^, Millipore^®^, United States) by using Trans-Blot Turbo^TM^ transfer system (Bio-Rad Laboratories, Germany). The membranes were blocked by incubating in blocking buffer (5% skim milk in PBST, PBST-PBS buffer containing 0.1% Tween 20), for 1 h at room temperature, washed three times with PBST and probed over night at 4°C with primary antibodies (β actin, cleaved PARP, cleaved caspase 3, cleaved caspase 8, cleaved caspase 9, Bcl 2, Bax and Glut 1 at 1:500 dilution). After washing three times with PBST for 5 min each, the membrane was incubated with horseradish peroxidase (HRP) conjugated secondary antibody at 1:1000 dilution and again washed three times in PBST. The bound antibodies were detected using an enhanced chemiluminescence substrate (Bio-rad, United States) and measured by densitometry using a ChemiDoc XRS digital imaging system and the Multi-Analyst software from Bio-Rad Laboratories (United States).

To ascertain the involvement of caspases 3 in morin induced cell death, downstream caspase 3 inhibitor was used. For this, cells were pretreated with caspase 3 inhibitor, z-DEVAD-fmk (30 μM) for 2 h prior to the addition of morin and cells were further treated with morin for 48 h (Mhaidat et al., 2014). After the incubation, cell viability was evaluated by MTT assay and the level of cleaved caspase 3 and cleaved PARP levels were examine by Western blotting as mentioned earlier.

### Measurement of Intracellular Reactive Oxygen Species (ROS) Generation

The effect of morin on intracellular ROS level was assessed using Fluorescent probe DCFH-DA staining (Cathcart et al., 1983). Initially cells were incubated with different concentrations of morin (150, 200, and 250 μM) for 48 h, washed with phosphate buffer saline (PBS, pH-7.4) then treated with DCFH-DA (20 μM) for 20 min and observed under fluorescent microscope (Pathway 855, BD Bioscience, United States) equipped with filters in the FITC range (Excitation, 490 nm; and Emission, 525 nm). For the exact measurement of ROS production cells were grown in 6 well plates and treated with indicated concentration of morin for 24 and 48 h. The harvested cells were rinsed twice with PBS, resuspended in 20 μM DCFH-DA and incubated at 37°C for 20 min in the dark. Data analysis was performed using Fluorescence Activated Cell Sorting (BD FACS Aria II, BD Biosciences, United States). The data acquisition and analysis were performed using BD FACSDiva^TM^ Software v6.1.2, and a minimum of 10000 cells was analyzed from each group.

### Antioxidant Assays

SW480 cells were pretreated with different concentrations of morin (150, 200, and 250 μM) for 48 h and positive control, H_2_O_2_ (200 μM) for 2 h. After incubation, cells were washed with PBS and lysed using respective enzyme specific buffer, and the lysed cells were used to determine the antioxidant activity. Cells without treatment were used as the control. The intracellular catalase activity was determined using catalase activity colorimetric assay kit according to the manufacturer instructions (K773-100) and the glutathione level was tested using glutathione assay kit according to the manufacturer instructions (K261-100).

### Measurement of Adenosine Tri Phosphate (ATP) Levels

The ATP levels in SW480 cells were determined using HPLC method (Hahn-Windgassen et al., 2005). After treatment, the cells were trypsinized and centrifuged at 800 × *g* for 3 min and the pellets were suspended in 4% perchloric acid on ice for 30 min. The pH of the lysate was adjusted between 6 and 8 with 2 M KOH. Precipitated salt was separated from the liquid phase by centrifugation at 13000 × *g* for 10 min at 4°C. ATP was quantified on a Prominence HPLC system (Shimadzu, Japan) containing LC-20 AD system controller, Phenomenex Gemini C18 column (250 mm × 4.6 mm, 5 μm), a column oven (CTO-20A), a Rheodyne injector (United States) with a loop of 20 μL volume and a diode array detector (SPD-M20A). A buffer 20 mM KH2PO4 and 3.5 mM K2HPO4 3H2O (pH 6.1) was used as the mobile phase. The flow rate was 1 ml/min, the injection volume was 20 μl and the column was at 37°C. The fractions were monitored at 259 nm. Sample peaks were identified by comparing with retention times of standard peaks. LC LabSolutions software was used for data acquisition and analysis.

### Fluorescence Analysis of 2-NBDG Uptake by Flow Cytometry

The changes in glucose uptake on treatment with morin (150, 200, and 250 μM) and camptothecin (50 μM) for 48 h, were examined using fluorescent D-glucose analog 2-[N-(7-nitrobenz-2-oxa-1,3-diazol-4-yl) amino]-2-deoxy-D-glucose (2-NBDG) followed by flow cytometric detection of fluorescence produced by the cells (Chen et al., 2010). Briefly, Cells grown in 6 well plates were treated with indicated concentration of morin for 48 h, the culture medium was replaced with 100 μM fluorescent 2-NBDG in PBS for 30 min, washed twice with cold phosphate-buffered saline (PBS), trypsinized, resuspended in ice-cold PBS and subjected to flow cytometry. Results were analyzed using Fluorescence Activated Cell Sorting (BD FACS Aria II, BD Biosciences, United States) at FITC range (excitation 490 nm, emission 525 nm band pass filter) and the mean fluorescence intensity of different groups were analyzed BD FACSDiva^TM^ Software v6.1.2 and a minimum of 10000 cells were analyzed from each group, corrected for auto fluorescence from unlabelled cells.

The effect of morin on Glut 1 protein expression was determined by Western blotting as explained earlier under immunoblot analysis.

### Statistical Analysis

Results were expressed as mean ± SD (standard deviation) from three independent experiments done in triplicates. The differences between treatments in comparison with control were assessed using one-way ANOVA and the significance of differences between means was calculated by Duncan’s multiple range test, using SPSS for Windows, standard version 16 (SPSS, Inc.), and significance was accepted at *p* ≤ 0.05.

## Results

### Morin Induces Cytotoxicity in Human Colorectal Cancer Cell Lines

**FIGURE 1:**
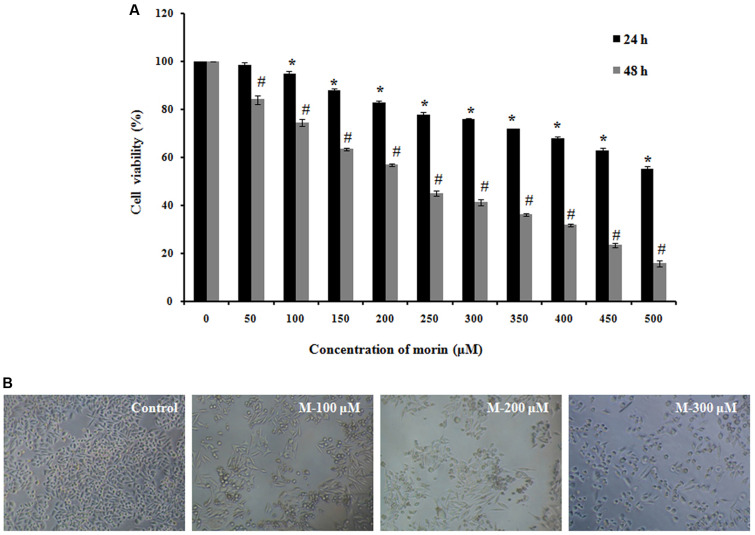
Morin induces cytotoxicity in SW480 colon cancer cells. **(A)** SW480 cells were treated with various concentrations of morin (50–500 μM) for 24 and 48 h. Cell viability was measured sing MTT assay. Results are shown as percentage reduction in cell viability of treated cells compared to untreated control cells. Values shown are the means ± SD obtained from three independent experiments. **(B)** Representative images when taken using phase contrast microscope. CAPT: camptothecin (50 μM), M (100, 200, and 300 μM): Morin (100, 200, and 300 μM). Significance levels between different groups were determined by using one way ANOVA, followed by Duncan’s multiple range test ^∗^*p* ≤ 0.05 versus control, 24 h; ^#^*p* ≤ 0.05 versus control 48 h.

MTT assay suggested that morin exhibits anticancer effects; the survival rate of the SW480 cells that were exposed to morin (50–500 μM) was reduced in a time and dose-dependent manner compared to the untreated control (**Figure 1A**). Analysis of variance results showed that the effects of dose and time on the viability of cells are statistically significant (*p* ≤ 0.05).

As shown in **Figure 1B**, the cellular morphology of SW480 was severely distorted, and cells became round on treatment with morin. Moreover, the cells showed a decline in number, indicating an increasing progression toward cell death. Meanwhile, the control cells displayed normal and healthy shapes.

### Morphological Changes in the Cells When Treated with Morin Using SEM

**FIGURE 2:**
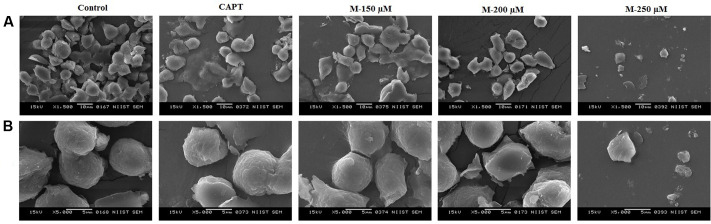
Scanning electron microscopic images of SW480 cells at 48 h after morin treatment. SW480 cells were treated with various concentrations of morin (150, 200, and 250 μM) and camptothecin (50 μM) for 48 h. After incubation, morphological changes were observed under SEM at magnification of 1500× **(A)** and 5000× **(B)** respectively.

The alterations in the surfaces of the cells after treatment with morin were observed under SEM (Lehmann et al., 2006). While the untreated control cells showed a smooth surface, treatment with morin resulted in severe damage of cells with ostensible deformation, shrunken to abnormal round type and the cell number was significantly decreased. At higher concentration of morin, we could observe separated apoptotic bodies as well as papillous protuberances on the surface of cells (**Figure 2**).

### Hoechst 33342 Staining

**FIGURE 3:**
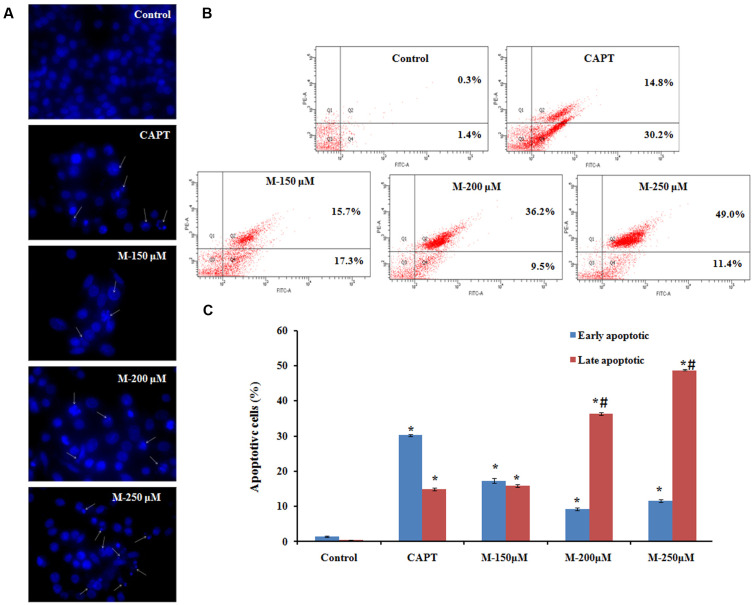
Effect of morin on apoptosis.SW480 cells were treated with various concentrations of morin (150, 200, and 250 μM) and camptothecin (50 μM) for 48 h. **(A)** Chromatin condensation and nuclear fragmentation observed using Hoechst 33342 staining, arrows represent cells with chromatin condensation inside the nucleus or fragmentation of the nucleus. **(B)** Annexin V-FITC/propidium iodide staining and flow cytometric analysis of cells undergoing apoptosis. **(C)** Flow cytometry analysis of the percentage of apoptotic cells in each group. CAPT: camptothecin (50 μM), M (150, 200, and 250 μM): Morin (150, 200, and 250 μM). Significance levels between different groups were determined by using one way ANOVA, followed by Duncan’s multiple range test. ^∗^*p* ≤ 0.05 versus control, ^#^*p* ≤ 0.05 versus camptothecin.

The antiproliferative activity shown by morin could be due to the induction of apoptosis and it can be observed by staining the cells with a fluorescent DNA-binding dye (DiBartolomeis and Mone, 2003). Nuclear alterations like chromatin condensation and DNA fragmentation which are the hallmarks of apoptosis were determined by Hoechst 33342 staining. As shown in **Figure 3A**, untreated control cells remained uniformly stained and emitted a blue fluorescence with consistent intensity, indicating that the chromatin was equivalently distributed in the nuclei. The fluorescence light was denser and brighter in cells treated with morin along with remarkable nuclear changes of apoptosis such as the formation of apoptotic bodies, condensation of chromatin and nuclear fragmentations. The number of apoptotic cells increased with the concentration of morin. Further confirmation of apoptosis in the cells was carried out by flow cytometry.

### Detection of Cell Apoptosis Using Flow Cytometry

Untreated and morin treated SW480 cells were stained with Annexin-V/FITC and propidium iodide (PI) followed by flow cytometry analysis for detecting phosphatidylserine (PS) externalization during apoptosis. Vital cells are negative for both fluorescence-conjugated annexin- V binding and in the case of propidium iodide uptake, early apoptotic cells are positive for fluorescence-conjugated annexin- V binding but negative for propidium iodide uptake, late apoptotic cells are positive for both, whereas necrotic cells are Annexin- V/FITC negative but PI positive (**Figure 3B**). The typical histogram representing cells in the early and late stage of apoptosis is illustrated in **Figure 3C**. A significant increase in the number of apoptotic cells was observed with the increase in the concentration of morin. The mean percentage of cells in the early apoptotic population and the late apoptotic population on treatment with 150 μM morin for 48 h was 17.26 ± 0.75 and 15.83 ± 0.41, respectively. It was further changed to 9.26 ± 0.40 and 11.56 ± 0.37 and 36.3 ± 0.42, 48.76 ± 0.20, respectively, after exposure to 200 and 250 μM morin, which was significantly different from control cell population (early apoptotic: 1.4 ± 0.2 and late apoptotic: 0.3 ± 0.1). The increment in the late apoptotic population on morin treatment (200 μM and 250 μM) were significantly higher than that of the positive control, camptothecin (early apoptotic: 30.2 ± 0.3 and late apoptotic: 14.8 ± 0.35).

### Morin Induced Apoptosis Is Associated with Loss of MMP (Δψm)

**FIGURE 4:**
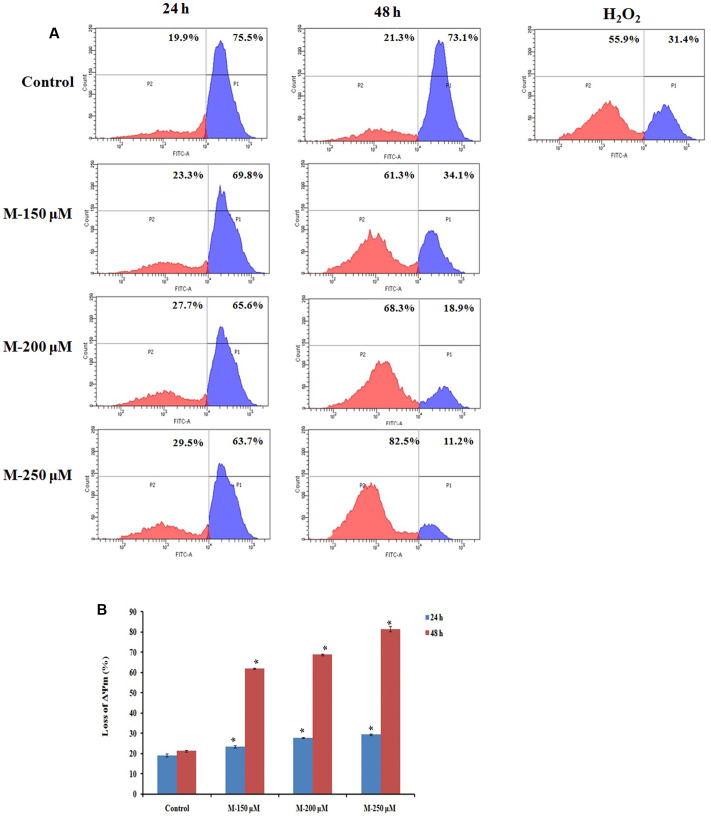
Quantification of loss of mitochondrial membrane potential by Rhodamine 123 staining. SW480 cells were treated with various concentrations of morin (150, 200, and 250 μM) for 24 and 48 h and H_2_O_2_ (200 μM) for 2 h. After incubation, cells were stained with Rhodamine 123 analyzed using flow cytometer. **(A)** Representative results. **(B)** Data analyzing fluorescence intensity from triplicate measurements. M (150, 200, and 250 μM): Morin (150, 200, and 250 μM). Significance levels between different groups were determined by using one way ANOVA, followed by Duncan’s multiple range test. ^∗^*p* ≤ 0.05 versus control.

It is evident from the above studies that apoptosis is induced in the SW480 cells on treatment with morin. As mitochondria play a vital role in the apoptotic cascade by serving as a convergent center of apoptotic signals for both intrinsic and extrinsic pathways, the changes induced in the mitochondrial membrane potential represent a determinant in the execution of cell death (Assuncao Guimaraes and Linden, 2004; Fleischer et al., 2006; Kim et al., 2006). Therefore, to assess the role of mitochondria in inducing apoptosis in SW480 cells, the change in mitochondrial membrane potential on morin treatment was examined. Mitochondrial membrane potential was assessed using rhodamine 123 dye. Rh123 enters only to mitochondria with an intact membrane potential and is retained in the mitochondria. Once the membrane potential is lost, the dye is leached out of the mitochondria and therefore a reduction in the fluorescence which is correlated with the mitochondrial membrane potential. The results indicated that the mitochondrial membrane potential of SW480 colon cancer cells depleted when pretreated with morin, in a dose and time-dependent manner (**Figure 4**). The mean percentage loss of mitochondrial membrane potential in SW480 cell, treated with 150, 200, and 250 μM morin, for 24 h was 23.4 ± 0.55, 27.76 ± 0.2, 29.43 ± 0.4, respectively, whereas the same after 48 h of treatment was 62 ± 0.3, 68.73 ± 0.37, 81.4 ± 1.2, respectively. As displayed in **Figure 4A**, when treatment time was increased from 24 to 48 h the loss of mitochondrial membrane potential was even higher than that of positive control H_2_O_2_. All the results were statistically significant compared to the corresponding untreated control groups (*p* ≤ 0.05).

### Morin Induced Apoptosis Is Associated with PARP Cleavage, Caspase Activation and Modulation of Bcl 2 Family Members (Bax and Bcl 2)

**FIGURE 5:**
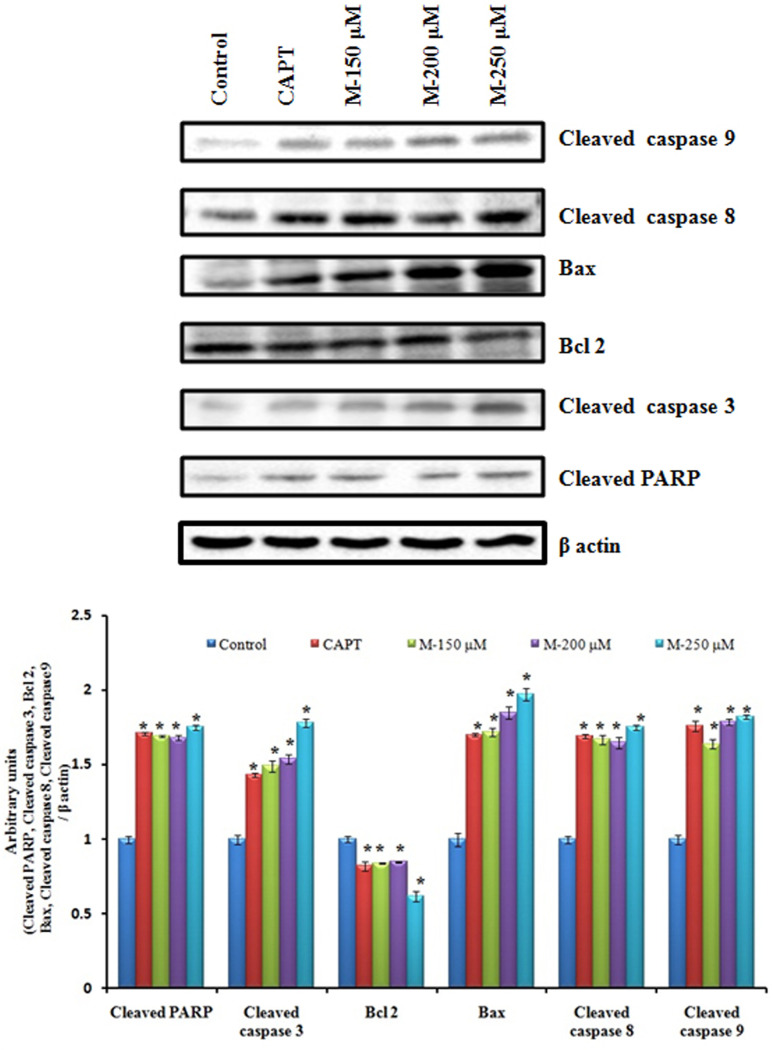
Effects of morin on the expression of apoptosis related proteins. SW480 cells were treated with various concentrations of morin (150, 200, and 250 μM) and camptothecin (50 μM) for 48 h. Western blot analysis for expression of cleaved PARP, cleaved caspase 3, Bcl2, Bax, Cleaved caspase 8 and cleaved caspase 9 was carried out. CAPT: camptothecin (50 μM), M (150, 200, and 250 μM): Morin (150, 200, and 250 μM).

Cleavage of PARP (Poly ADP-Ribose Polymerase), a nuclear enzyme having a significant role in DNA repair by caspases has been considered as a hallmark of apoptosis. An increase in the concentration of cleaved PARP as well as cleaved caspase 3 was observed to be increased in morin treated cells compared to the untreated control cells. To investigate the pathway by which morin induce apoptosis further, levels of cleaved caspase 8, cleaved caspase 9 were evaluated. An up-regulation of the levels of both the caspases were observed indicating morin is inducing apoptosis via both extrinsic and mitochondria-mediated (intrinsic) pathways. The western blot analysis for Bax and Bcl 2 were carried out to determine whether morin induced apoptosis occurred through alterations in expression of the pro and anti-apoptotic proteins. It was found that the expression of Bax increased while Bcl 2 expression was reduced on treatment with morin (**Figure 5**).

**FIGURE 6:**
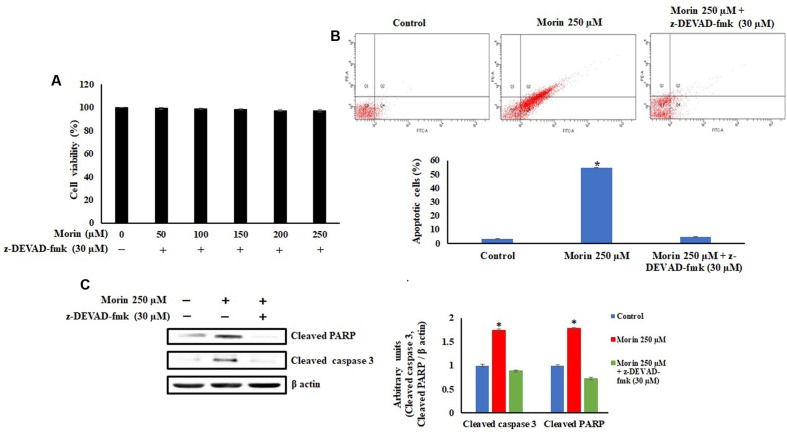
Morin induces caspase 3 mediated apoptosis. SW480 cells were pretreated with the caspase 3 inhibitor, z-DEVAD-fmk (30 μM), for 2 h prior to the addition of morin and cells were further incubated for 48 h. **(A)** Change in cell viability was assessed by MTT assay, **(B)** Apoptosis was measured using Annexin V-FITC/propidium iodide staining and flow cytometric analysis and **(C)** level of cleaved caspase 3 and cleaved PARP levels were examine by Western blotting. Each value represents mean ± SD (standard deviation) from three independent experiments. Significance levels between different groups were determined by using one way ANOVA, followed by Duncan’s multiple range test. ^∗^*p* ≤ 0.05 versus control.

As the caspase activation pathway has been known to play a key role in the execution of apoptosis, we confirmed relationship between the apoptotic cell death induced by morin and activation of downstream caspases by using its specific inhibitor. A reduction in cell death was observed with a corresponding decrease in cleaved caspase 3 and cleaved PARP (**Figure 6**) indicating the role of morin in the event.

### Morin Induced Loss of MMP (Δψm) and Apoptosis Is Mediated via ROS Formation

**FIGURE 7:**
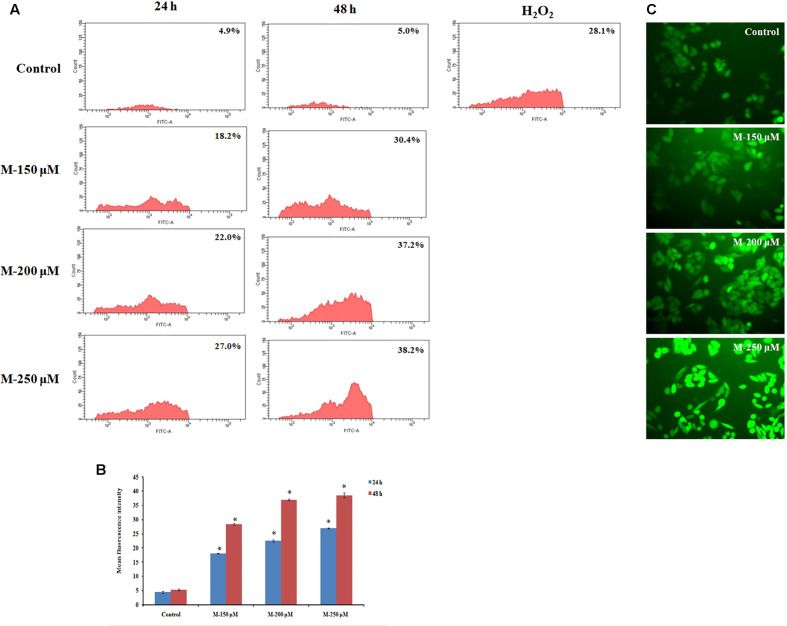
Quantification of ROS formation by DCFH-DA staining. SW480 cells were treated with various concentrations of morin (150, 200, and 250 μM) for 24 and 48 h and H_2_O_2_ (200 μM) for 2 h. After incubation, cells were stained with DCFH-DA and analyzed using flow cytometer. **(A)** Representative results. **(B)** Data analyzing fluorescence intensity from triplicate measurements. **(C)** Fluorescent imaging using fluorescent microscope. M (150, 200, and 250 μM): Morin (150, 200, and 250 μM). Significance levels between different groups were determined by using one way ANOVA, followed by Duncan’s multiple range test. ^∗^*p* ≤ 0.05 versus control.

It is reported that cancer cells are with increased oxidative stress and are more sensitive and susceptible to exogenous agents induced a rapid surge in ROS levels (Nogueira and Hay, 2013). The ROS level in the cells on treatment with morin was determined by measuring the intracellular ROS levels, by detecting dichlorofluorescein (DCF), derived from the oxidation of H_2_DCFDA by ROS, using flow cytometry as well as the fluorescent microscope. The results indicated a dose and time-dependent increase in ROS production in the cells (**Figure 7**) on pretreatment with morin. The mean percentage increase in ROS levels in SW480 cell, treated with 150, 200, and 250 μM morin, for 24 and 48 h, determined by DCFH-DA staining followed by flow cytometry was 18.03 ± 0.15, 22.5 ± 0.43, 27 ± 0.2 and 28.36 ± 0.3, 36.9 ± 0.26, 38.5 ± 0.88, respectively and were statistically significant compared to corresponding control groups (*p* ≤ 0.05).

### Morin Reduced Antioxidant Level in SW480 Cells

**FIGURE 8:**
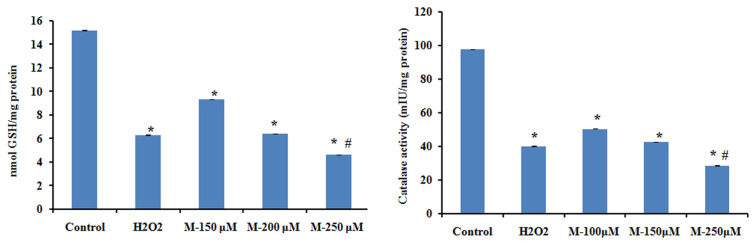
The levels of reduced glutathione and catalase activity were assayed after treatment with of morin (150, 200, and 250 μM) for 48 h and H_2_O_2_ (200 μM) for 2 h. Each value represents mean ± SD (standard deviation) from triplicate measurements. CAPT: camptothecin (50 μM), M (150, 200, and 250 μM): Morin (150, 200, and 250 μM). Significance levels between different groups were determined by using one way ANOVA, followed by Duncan’s multiple range test. ^∗^*p* ≤ 0.05 versus control, ^#^*p* ≤ 0.05 versus camptothecin.

To check the changes in the antioxidant status in SW480 cell on morin treatment, catalase activity and glutathione levels were analyzed. It is reported that ROS-mediated apoptotic signaling is associated with decreased cellular GSH levels either by ROS-induced GSH oxidation or by GSH export from cells (Lu and Armstrong, 2007). ROS-dependent apoptotic effect is also linked with inactivation of intracellular catalase that may contribute to the efficiency of ROS mediated intercellular induction of apoptosis. The results showed a significant decrease in the levels of both catalase and glutathione in the cells, in a dose-dependent manner when compared with untreated control cells (*p* ≤ 0.05). On treatment with 250 μM morin, the decrease was even significantly higher than that of positive control used (*p* ≤ 0.05) (**Figure 8**).

### Morin Treatment Resulted in Significant Reduction in ATP Level with Corresponding Increase in ADP and AMP Levels

**FIGURE 9:**
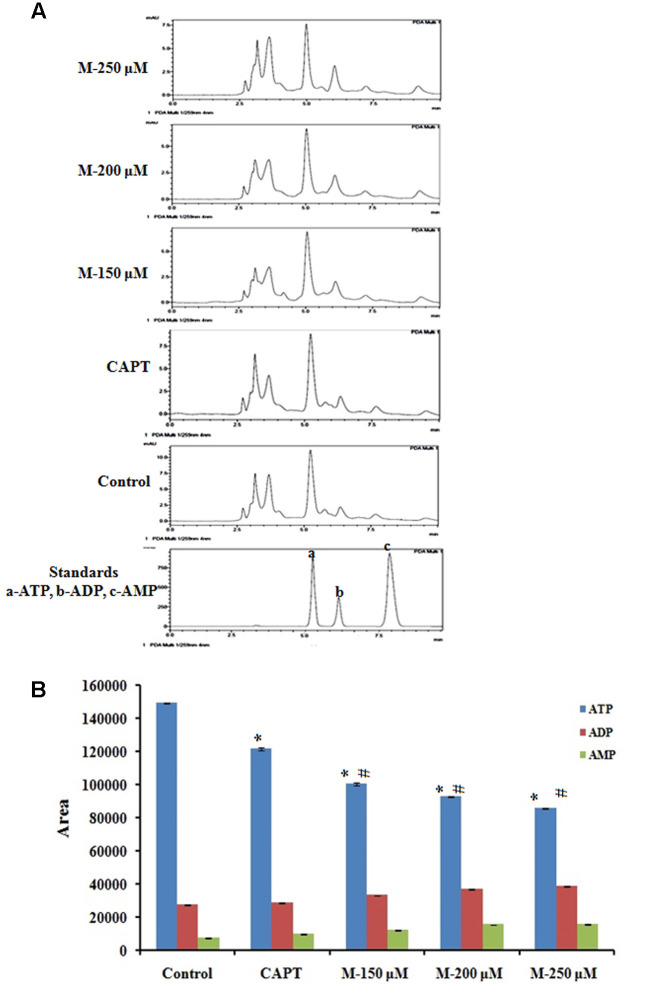
Quantification of ATP level by HPLC method. SW480 cells were treated with various concentrations of morin (150, 200, and 250 μM) and camptothecin (50 μM) for 48 h. Morin treatment significantly decreases mitochondrial capacity to produce ATP. **(A)** Representative HPLC chromatogram showing decrease in ATP production in SW480 cells on treatment morin. **(B)** Graphical representation of reduction in ATP production by morin in SW480 colon cancer cells. CAPT: camptothecin (50 μM), M (150, 200, and 250 μM): Morin (150, 200, and 250 μM). Each value represents mean ± SD from triplicate measurements and significance levels between different groups were determined by using one way ANOVA, ^∗^*p* ≤ 0.05 versus Control; ^#^*p* ≤ 0.05 versus camptothecin.

Since ATP is the primary energy currency of the cell and uncontrollably dividing cancer cells have a high demand for ATP, we tried to understand the effect of morin on cellular ATP levels. Cellular ATP levels were determined by HPLC method and results showed (**Figure 9**) morin pretreatment resulted in a significant decrease in ATP level with a simultaneous increase in ADP and AMP levels when compared with untreated control cells (*p* ≤ 0.05).

### Decreased ATP Levels in Morin Treated SW480 Cells Are Associated with Reduced Glucose Uptake and Glut 1 Expression

**FIGURE 10:**
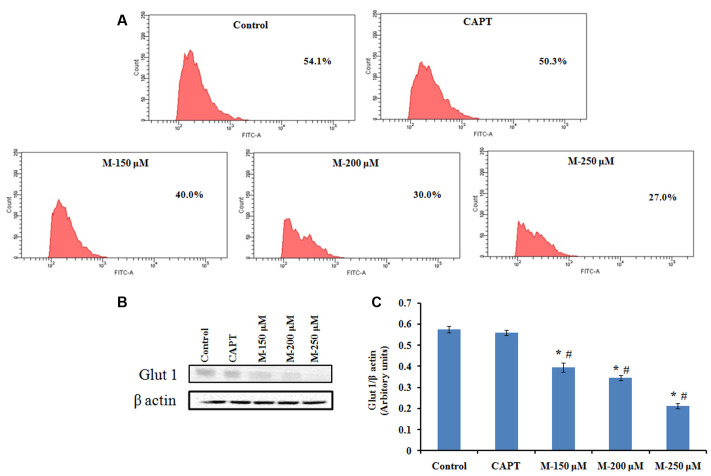
Flow cytometry analysis of 2-NBDG uptake and western blot analysis for Glut 1 expression. SW480 cells were treated with various concentrations of morin (150, 200, and 250 μM) and camptothecin (50 μM) for 48 h. **(A)** FACS analysis of 2-NBDG uptake in SW480 cells plotting cell count against FITC. **(B)** Glut 1 expression analyzed by western blotting. **(C)** Quantification of Glut 1 protein levels. CAPT: camptothecin (50 μM), M (150, 200, and 250 μM): Morin (150, 200, and 250 μM). Each value represents mean ± SD (standard deviation) from three independent experiments. Significance levels between different groups were determined by using one way ANOVA, followed by Duncan’s multiple range test. ^∗^*p* ≤ 0.05 versus control, ^#^*p* ≤ 0.05 versus camptothecin.

Cancer cells are reported to use glucose for aerobic glycolysis and their need for high amount of glucose will lead to augmentation of glycolytic metabolism and increase in the glucose transport across the plasma membrane (Brown and Giaccia, 1998; Macheda et al., 2005). Compared to the normal cells, cancer cells demonstrate increased sensitivity to glucose deprivation-induced cytotoxicity (Aykin-Burns et al., 2009). Glut 1 is the natural transporter of glucose and is required for the high glycolytic rate seen in colorectal tumors. In support to this, in the present study, it was noticed that glucose uptake and Glut 1 expression was reduced significantly in the cells pretreated with morin (**Figure 10**) when compared to the untreated control cells (*p* ≤ 0.05).

## Discussion

The increasing need to develop anti-cancer agents with lesser cytotoxicity and side effects has led the investigators to explore new sources of pharmacologically active compounds from natural products with a broad range of antitumor mechanisms. It is reported that the intake of fruits, vegetables, medicinal herbs and their extracts have positive effects in prevention and control of cancer compared with currently available treatment approaches like chemotherapy or new hormonal therapies (Wang et al., 2010). So, in the present study we have focused on elucidating the anticancer effect and underlying mechanism of morin, a major flavonoid found in the Moraceae family members in SW480 colon cancer cells.

Cell viability assay showed that morin exhibited a chemopreventive effect on SW480 cells in a time and dose-dependent manner with associated morphological changes. Apoptosis is a fundamental mechanism by which cells undergo death to control cell proliferation. Interference in the innate apoptotic activity is considered as a hallmark of neoplastic transformation and tumor formation. Modulation of the apoptotic cascade has been proposed as an innovative approach for the treatment of cancer (Kaufmann and Earnshaw, 2000; Ghobrial et al., 2005). In anticancer therapy, compounds that can actuate apoptosis have been considered to possess anticancer potential (Kelly and Strasser, 2011; Strasser et al., 2011). In the present study, typical apoptotic characteristics were observed under SEM and the highly condensed nucleus or fragmented chromatin that was uniformly fluorescent as observed by fluorescence microscopy in cells treated with morin by Hoechst 33342 staining indicated that morin could induce apoptosis. The externalization of phosphatidylserine during apoptosis was further confirmed with Annexin- V/FITC and PI staining followed by flow cytometry analysis.

Further studies were conducted to explore the mechanism by which morin induced apoptosis in SW480 cells. Mitochondria are considered as a novel target for chemotherapy-induced apoptosis in the recent times. Loss of mitochondrial membrane potential due to opening of mitochondrial permeability transition pores is an indicative of apoptosis and in the current study, a significant loss of mitochondrial membrane potential was observed in SW480 cells on morin treatment (Hengartner, 2000; Kim et al., 2007). The highly complex process of the energy dependent cascade of molecular events in apoptosis is mainly mediated through two primary linked apoptotic pathways, the extrinsic and the intrinsic pathways. The extrinsic pathway accompanied by activation of initiator caspase 8 via death receptors on the cell surface. The intrinsic pathway acts via apoptotic cascades mitochondrion, which results in the amendment of the membrane potential (ΔΨm) and the activation of initiator caspase 9 (Chowdhury et al., 2006). Both initiator caspases can activate downstream caspase 3 and lead to cleavage of the PARP protein, which commits cells to apoptosis (Tewari et al., 1995; Woo et al., 2011). In the current study treatment of SW480 cells with morin resulted in caspase 3 mediated PARP cleavage by the activation both extrinsic as well as intrinsic pathways of apoptosis which was ascertained by using the downstream caspase 3 inhibitor. There was a reduction in the cell death when cells were treated with downstream caspase 3 inhibitor before exposing to morin, indicating the role of morin in inducing the cell death. It was also observed that the levels of cleaved caspase 3 and cleaved PARP were significantly reduced on pretreating SW480 cells with caspase 3 specific inhibitor, which confirmed that the caspase 3 activation plays a significant role in morin induced cell death in SW480 colon cancer cells.

Members of the Bcl 2 family function as key regulators of mitochondrial response to apoptotic signals. Increased ratio of antiapoptotic to proapoptotic Bcl 2 proteins is reported in most of the neoplastic cells and which enables them to survive under adverse conditions. An upcoming approach for cancer therapy is by direct activation of the apoptotic pathway by enhancing the function of proapoptotic Bcl 2 proteins and reducing the activity of antiapoptotic Bcl 2 proteins (Certo et al., 2006; Vogler et al., 2009). Thus activation of Bax in tumor cells could be an effective treatment strategy (Walensky and Gavathiotis, 2011). In the present study when SW480 cells were treated with morin, an upregulation of Bax protein level and downregulation of Bcl 2 protein level was observed and which further confirms the anticancer potential of morin.

Compared to the normal cells, cancer cells are more sensitive to rapid increases in ROS levels and ROS released during several conventional treatments can mediate proapoptotic effects in cancer cells (Engel and Evens, 2006; Gallego et al., 2008). Cancer cells with high levels of antioxidant systems and mitochondrial suppressor of ROS (e.g., uncoupling protein-2), have been found to induce chemoresistance in them (Ramanathan et al., 2005; Derdak et al., 2008). Hence the modulation of oxidative stress in tumor cells has been suggested as a noteworthy approach to sensitize tumors to cytotoxic drugs. The existence of an excellent balance between intracellular ROS levels and ROS scavenging antioxidant systems is maintaining the redox homeostasis in cells. During oxidative stress, this balance is disturbed as seen in most of the cancer cells which make them depend on their antioxidant system to maintain redox balance and hence they are more susceptible to further oxidative stress. Therefore, any agent that augments intracellular ROS level in cancer cells to a toxic level can result in mitochondrial damage and cell death (Pramanik et al., 2011). In the current study, an increased ROS level along with reduced antioxidant status in morin treated cells point out that morin is inducing ROS-mediated apoptosis in SW480 colon cancer cells which were in agreement with the study done by Hyun et al. (2015).

Cells can produce energy either via mitochondria-dependent pathways (electron transport chain and Kreb cycle) or glycolysis. Warburg has reported that compared to normal cells, aerobic glycolytic activity is augmented in cancer cells by increasing the glucose transportation into the cytoplasm and limiting downstream mitochondrial respiration (Warburg, 1956). The entry of glucose into the cell occurs by facilitated diffusion and is mainly dependent on glucose transporters and hence its inhibition represents a very effective way of preventing cancer proliferation. Elevated Glut1 expression (Chung et al., 2009; Saigusa et al., 2012) and high insulin levels (Wolpin et al., 2009) have been associated with CRC stage of poor prognosis and Glut 1 has been considered as a potential therapeutic target to control the glucose uptake by the cells to limit the proliferative capacity of the CRC cells. A study by Aguilera et al. (2016) has shown that vitamin C uncouples the Warburg metabolic switch in KRAS mutant colon cancer by strong downregulation of the glucose transporter Glut 1. Our results from glucose uptake study have shown that there was a significant decline in cellular glucose uptake on treatment with morin in a dose-dependent manner along with significant reduction in Glut 1 expression and cellular ATP level. Thus, morin treatment resulted in impairment in mitochondrial functioning as well as reduced glucose availability/metabolism leading to an energetic stress and finally to death of the cancer cells.

Earlier studies suggest plant derived phytochemicals can modulate Warburg effect and hence the cancer potential. Among various phytochemicals genistein and fasentin target glucose transporter Glut 1 and exert antitumor effects by inhibiting glucose uptake in tumor cells, thus leading to glucose deprivation mediated cell death (Boros et al., 2001). Curcumin (Liao et al., 2015), resveratrol (Gwak et al., 2015) and plumbagin (Sinha et al., 2013) is also reported to down-regulate Glut 1. Epigallocatechin gallate (Moreira et al., 2013) and honokiol (Wu et al., 2010) have also been reported to alter glucose metabolism leading to anticancer effects. Various critical enzymes that are involved in aerobic glycolysis, have also been exploited as targets for cancer therapies. Methyl jasmonate inhibit hexokinase (Rotem et al., 2005), gossypol acts as a lactate dehydrogenase inhibitor (Gomez et al., 1997) and cinnamic acid derivatives act as class of monocarboxylate transporter inhibitors (Halestrap and Denton, 1974) are the most studied among them. These studies suggest that there is immense potential to develop anticancer drugs based on the relationship between aerobic glycolysis and cancer progression.

## Conclusion

In the present study, SW480 colon cancer cells were treated with morin at different concentrations that induced formation of reactive oxygen species in the cells. The formation of ROS led to the disturbance in the mitochondrial functioning resulting in intrinsic as well as the extrinsic pathway of apoptosis. It was also observed that morin could restrict entry of glucose into the cells by recuing Glut 1 expression. The inhibition of Glut 1 represents a potent way of attacking cancer by blocking its main nutrient uptake resulting in reduction of the glycolytic flux which further sensitizes cells to undergo mitochondria dependent apoptosis. Therefore, in the present study pro-oxidant action as well as uncoupling of Warburg effect together contributed to the anticancer activity of morin in SW480 colon cancer cells. These evidences suggest that morin may be a promising therapeutic agent against colorectal cancer.

## Author Contributions

TS carried out the experiments, executed the work and prepared the manuscript. KA, HS, and TR were associated in various experiments. PN, conceptualized the work, planed the experiments and corrected the manuscript.

## Conflict of Interest Statement

The authors declare that the research was conducted in the absence of any commercial or financial relationships that could be construed as a potential conflict of interest. The reviewer LV and handling Editor declared their shared affiliation.
