# Corrigendum: Morin inhibits proliferation of SW480 colorectal cancer cells by inducing apoptosis mediated by reactive oxygen species formation and uncoupling of Warburg effect

**DOI:** 10.3389/fphar.2025.1580299

**Published:** 2025-03-27

**Authors:** Thomas Sithara, K. B. Arun, H. P. Syama, T. R. Reshmitha, P. Nisha

**Affiliations:** ^1^ Agro Processing and Technology Division, National Institute for Interdisciplinary Science and Technology (CSIR), Thiruvananthapuram, India; ^2^ Academy of Scientific and Innovative Research, New Delhi, India

**Keywords:** colorectal cancer, reactive oxygen species, apoptosis, Warburg effect, energetic stress

In the published article, there was an error in the text values.

A correction has been made to **Results**, *Detection of Cell Apoptosis Using Flow Cytometry*. This sentence previously stated:

“The mean percentage of cells in the early apoptotic population and the late apoptotic population on treatment with 150 µM morin for 48 h was 17.26 ± 0.75 and 9.26 ± 0.40, respectively. It was further increased to 11.56 ± 0.37, 15.83 ± 0.41 and 36.3 ± 1.35, 48.76 ± 0.2 after exposure to 200 and 250 µM morin, respectively, which was significantly different from control cell population (early apoptotic: 1.4 ± 0.2 and late apoptotic: 0.3 ± 0.1). The increment in the late apoptotic population on morin treatment (250 µM) was significantly higher than that of the positive control, camptothecin (early apoptotic: 14.8 ± 0.12 and late apoptotic: 30.2 ± 0.18)”.

The corrected sentence appears below:

“The mean percentage of cells in the early apoptotic population and the late apoptotic population on treatment with 150 µM morin for 48 h was 17.26 ± 0.75 and 15.83 ± 0.41, respectively. It was further changed to 9.26 ± 0.40 and 11.56 ± 0.37 and 36.3 ± 0.42, 48.76 ± 0.20, respectively, after exposure to 200 and 250 µM morin, which was significantly different from control cell population (early apoptotic: 1.4 ± 0.2 and late apoptotic: 0.3 ± 0.1). The increment in the late apoptotic population on morin treatment (200 µM and 250 µM) were significantly higher than that of the positive control, camptothecin (early apoptotic: 30.2 ± 0.3 and late apoptotic: 14.8 ± 0.35).”

The authors apologize for this error and state that this does not change the scientific conclusions of the article in any way. The original article has been updated.

